# Part I, Patient perspective: activating patients to engage their providers in the use of evidence-based medicine: a qualitative evaluation of the VA Project to Implement Diuretics (VAPID)

**DOI:** 10.1186/1748-5908-5-23

**Published:** 2010-03-18

**Authors:** Stacey A Pilling, Monica B Williams, Rachel Horner Brackett, Ryan Gourley, Mark W Vander Weg, Alan J Christensen, Peter J Kaboli, Heather Schacht Reisinger

**Affiliations:** 1 The Center for Research in the Implementation of Innovative Strategies in Practice (CRIISP), Iowa City VA Medical Center, 601 Hwy 6 West, Mail Stop 152, Iowa City, IA, 52246-2208, USA; 2 Division of General Medicine, Department of Internal Medicine, University of Iowa Carver College of Medicine, Iowa City, IA, USA; 3 Department of Psychology, University of Iowa, Iowa City, IA, USA

## Abstract

**Background:**

This qualitative evaluation follows a randomized-control trial of a patient activation intervention in which hypertensive patients received a letter in the mail asking them to discuss thiazide diuretics with their provider. Results of the parent study indicated that the intervention was effective at facilitating discussions between patients and providers and enhancing thiazide prescribing rates. In the research presented here, our objective was to interview patients to determine their receptivity to patient activation, a potential leverage point for implementing interventions.

**Methods:**

Semi-structured phone interviews were conducted with 54 patients, purposefully sampled from a randomized controlled trial of a patient activation intervention. All subjects had a history of hypertension and received primary care from one of twelve Veterans Affairs primary care clinics. All interviews were transcribed verbatim and reviewed by the interviewer. Interviews were independently coded by three qualitative researchers until consensus was attained, and relevant themes and responses were identified, grouped, and compared. NVivo 8.0 was used for data management and analysis.

**Results:**

Data from this qualitative study revealed that most participants held favorable opinions toward the patient activation intervention used in the clinical trial. Most (82%) stated they had a positive reaction. Patients emphasized they liked the intervention because it was straightforward and encouraged them to initiate discussions with their provider. Also, by being active participants in their healthcare, patients felt more invested. Of the few patients offering negative feedback (11%), their main concern was discomfort with possibly challenging their providers' healthcare practices. Another outcome of interest was the patients' perceptions of why they were or were not prescribed a thiazide diuretic, for which several clinically relevant reasons were provided.

**Conclusion:**

Patients' perceptions of the intervention indicated it was effective via the encouragement of dialogue between themselves and their provider regarding evidence-based treatment options for hypertension. Additionally, patients' experiences with thiazide prescribing discussions shed light on the facilitators and barriers to implementing clinical practice guidelines regarding thiazides as first-line therapy for hypertension.

**Trial registration:**

National Clinical Trial Registry number NCT00265538

## Background

A communication gap frequently exists between physicians and patients regarding their healthcare decisions. Multiple studies have found physicians often assume a paternalistic role in their healthcare management of patients [1-5]. In these types of interactions, patients tend to delegate all decision-making power to their providers, refraining from both expressing their concerns during medical visits and from asking questions pertaining to their healthcare [6-8]; consequently, an exchange of information between patient and provider is limited. However, as recent studies have demonstrated, when patients and providers establish a collaborative relationship, that considers patient contributions and preferences, treatment may be more effective [2,9-13].

One method of establishing this bidirectional therapeutic decision-making process is by means of patient-activated interventions [2-4,14]. Such interventions work to increase patient involvement in personal healthcare through patient education and skill-building, often targeted toward patients initiating specific conversations with their providers. These interventions promote a bidirectional interaction between providers and patients. A critical element in this model is how to motivate patients to inquire about or request the promoted therapy or service. One approach widely utilized in marketing is direct-to-consumer (DTC) advertising via mass media portals, including television and magazine advertisements and personalized direct mail [15]. While DTC advertising is considered controversial in the medical field [10,15,16], it can serve as a useful tool for consumers to become active team members in the management of their healthcare. Patient activation seeks to utilize elements of direct-to-consumer advertising by incorporating aspects of social marketing [17] to promote evidence-based therapies rather than brand-specific pharmaceuticals. These interventions could be a critical component of implementing guideline-concordant therapy in a consumer-driven healthcare approach [18,19], yet little is known about patients' receptivity to such an intervention.

In this paper, we describe patient perspectives of a patient activation intervention to encourage patients to talk with their primary care provider about initiating clinical practice guideline-concordant therapy (*i.e*., thiazide diuretics) for hypertension. A complementary paper examines provider perspectives of the same intervention [20]. These patients and providers were part of a randomized control trial that found the intervention increased the likelihood of patients discussing a thiazide diuretic with their provider and the likelihood that providers would prescribe the medication [21]. Semi-structured interviews were conducted with two stakeholder groups--patients and primary care providers--to give them the opportunity to evaluate the effectiveness of the intervention from their own point of view. These two qualitative studies are first steps in systematically examining how to modify this patient activation intervention to more effectively engage all involved. The provider study places the intervention in the larger context of strategies to implement clinical practice guidelines with a focus on hypertension and prescribing behavior. The patient study looks more closely at how patients viewed their role in the intervention as initiators of guideline-concordant therapy. Together they provide a more comprehensive picture of how the intervention worked in practice and point to ideas to improve its effectiveness for future implementation studies and interventions.

The primary objective of this study is to determine participants' perceptions of a patient-activated intervention, particularly its acceptability and effectiveness through the eyes of the patients. Understanding patients' receptivity to this approach, their motivations for participation, and their perspective of their roles in the intervention may improve the implementation of patient activation strategies to promote clinical practice guidelines, as well as enhancing a collaborative approach between patients and providers.

## Methods

### Participants, intervention, and recruitment

We conducted semi-structured interviews with 54 veterans with hypertension, recruited from a larger group of 532 veterans participating in a hypertension study at the Veterans Affairs Medical Centers (VAMCs) in Iowa City, IA and Minneapolis, MN [22]. All patients in the study received primary care at one of these two facilities or through one of their community-based outpatient clinics (five in IA, five in MN). The parent study involved a randomized controlled trial of a patient activation intervention to encourage hypertensive patients to speak with their provider about obtaining a prescription for a thiazide diuretic, first-line therapy for hypertension. The objective of the parent study was to change provider prescribing behavior and increase implementation of clinical practice guidelines. Patients were randomized to a control arm or one of three intervention arms who received: (arm A) an individualized letter discussing their latest blood pressure, their 10-year cardiovascular risk score, and education about the value of thiazides; (arm B) the same individualized letter plus an offer of a $20 financial incentive if they talked with their provider about a thiazide prescription, and, if applicable, a copayment reimbursement for six months ($48) if prescribed a thiazide; and (arm C) the individualized letter, the financial incentive, plus a phone call from a health educator to answer questions about the intervention. Patients were asked to return a postcard (themselves or by giving it to their provider to complete) indicating whether they talked with their provider about their hypertension, whether they were prescribed a thiazide diuretic, and, if not, their understanding of their provider's rationale for not initiating thiazide treatment.

Patients for the semi-structured interviews were recruited according to a purposeful stratified sampling design by site (IA or MN), intervention arm (A, B, C), and whether or not they were prescribed a thiazide diuretic. Patients were identified as being prescribed a thiazide diuretic or not through review of the electronic medical record. The study team also attempted to interview all patients who returned a postcard stating they chose not to bring in the letter (n = 7). We conducted these interviews outside the stratified sampling design to gain more insight into why patients chose not to participate in the intervention. We completed three of the possible seven interviews with patients who indicated on the postcard that they chose not to bring in the letter. The other four were unable to be reached for follow-up.

### Interviews

Open-ended, semi-structured interview guides were developed for each arm of the study to cover variation in patient activation strategies. A semi-structured approach was chosen to minimize variation among interviewers and to facilitate a systematic means of gathering data and conducting analysis of responses, while at the same time allowing for individualized follow-up depending on the content of the interview [23]. Interview guides were evaluated and revised periodically throughout the study period as analysis evolved and new themes emerged. During the interviews, lasting an average of 16 minutes (range: 9-46 minutes), veterans were asked about their: opinions of the intervention, factors affecting their decision regarding whether or not to bring in the letter, conversation with their healthcare provider about thiazides, understanding of why they were or were not prescribed a thiazide, and opinions of financial incentives. Field notes were completed immediately after each interview using a standardized template.

After conducting the first set of interviews, the lead qualitative researcher (HSR) trained two research assistants (RHB, MW) to conduct the subsequent interviews. The interviews were conducted within two weeks of the primary care visit in which patients were asked to bring in the letter. One exception was patients who sent in postcards marked 'no I did not talk to my doctor.' These interviews were attempted as soon as the qualitative interviewers were made aware of their return. The interviews took place over a nine-month period from March to December 2007. All interviews were completed by telephone, except for one completed in person, and recorded on digital voice recorders. Interviews were transcribed verbatim and reviewed against the original recording by the interviewer prior to importation into NVivo 8, a qualitative data management and analysis software program [24]. Interviewer field notes were also imported into NVivo for analysis and comparison. The study was approved by the Institutional Review Boards of the Iowa City and Minneapolis VAMCs and the respective VA Research and Development Committees.

### Analysis

Coding analysis consisted of three stages: data collection and thematic content analysis occurring simultaneously; detailed analysis of the thematic codes; and matrix analysis of codes. The first stage was an iterative process with coding analysis and data collection occurring simultaneously and informing both the evolving interview guide and coding dictionary [25-27]. After conducting the first set of interviews, the lead qualitative researcher (HSR) and a research assistant (MW) read through the transcripts, made notes on preliminary coding, and developed a thematic coding scheme with definitions. This coding 'dictionary' was routinely reviewed and refined throughout the data collection process as new themes emerged. After the initial dictionary was developed, each transcript was independently coded by a minimum of three individuals from the research team (HSR and trained research assistants, RHB, MW, and/or RG). They then met as a team to compare their impressions and code the transcript by consensus within NVivo. Weekly consensus coding was performed in an effort to increase the validity and reliability of the coding by refining the content boundaries of the codes and making coding more consistent. An audit trail was kept in NVivo. At the end of the data collection and analysis, the coding dictionary contained 18 thematic codes. As new codes were added to the coding dictionary, previous transcripts were coded for content related to the new codes.

In the second coding stage, detailed analysis of the original thematic codes was performed and content sub-coded into related subcategories [23] resulting in 31 additional sub-codes. In stage three, matrix analyses [28] of a set of sub-codes focused on several specific questions, including: 'What did you think about the letter and what it asked you to do?' 'What made you decide to take the letter with you to your appointment rather than just leaving it at home?' 'What would you say was the main reason that you were (not) prescribed a diuretic?' Two coders (SP, MW) independently coded each participant's response to the questions based on the specific question. For example, the question regarding participants' opinions of the intervention letter were coded to one of the following mutually exclusive categories: positive, neutral, negative, or no response. Disagreements were resolved through a third coder (HSR) who acted as a tiebreaker. These questions were coded by mutually exclusive discreet categories to allow for a structured presentation of the distribution of responses of the participants. To maintain consistency, only responses patients gave directly after the question was asked were coded.

## Results

The mean age of our study sample was 65.1 years, 98% were male, and 76% had a copayment for their medications (Table 1). Demographic and baseline characteristics were similar between those included in the qualitative study and the larger study sample. The one notable difference between the groups was the higher proportion of semi-structured interview participants were prescribed a thiazide. This difference was intentional due to the decision to stratify by prescription outcome in an effort to better understand the main outcome of the parent study. Results of the parent study indicate the intervention was effective at facilitating discussions between patients and providers and enhancing thiazide prescribing rates [21].

**Table 1:** Characteristics of intervention and qualitative samples at index visit

	Total Intervention Sample (N = 478)	Qualitative Sample (N = 54)	P-value
Age (years)	64.0	65.1	0.38

Gender (male)	472 (98.7%)	53 (98.1%)	0.53

Site (IA)*	279 (58.4%)	29 (53.7%)	0.56

Co-pay for medications	336 (70.3%)	41 (75.9%)	0.63

Systolic BP (mmHg) (Goal: <140 or < 130)	135.1	138.2	0.15

Diastolic BP (mmHg) (Goal: <90 or <80)	78.1	79.4	0.45

At BP Goal	214 (44.8%)	26 (48.1%)	0.67

Intervention Arm*			0.73

Arm A	175 (36.6%)	18 (33.3%)	

Arm B	144 (30.1%)	19 (35.2%)	

Arm C	159 (33.3%)	17 (31.5%)	

Prescribed Thiazide*	112 (23.4%)	26 (48.1%)	<0.0001

*Qualitative sample stratified by these variables.

### Patient perceptions of intervention

A critical component of the qualitative evaluation was to determine patients' perceptions of the intervention and what motivated them to bring the letter to their provider. A majority of patients (82.9%) believed the letter was a positive instrument for initiating discussion with their providers pertaining to their hypertension (Table 2). The semi-structured interviews offered insight and presented common themes that helped elucidate the factors that contribute to the effectiveness and acceptability of patient activation as an intervention strategy.

**Table 2:** Patients' reported perception of letter

Opinion of Letter	(N = 41)^
Positive*	34 (82.9%)

Neutral	1 (2.4%)

Negative	4 (9.8%)

Did not remember letter	2 (4.9%)

^Thirteen participants did not have complete question/response pairs (participant was not asked the question or did not directly answer it when asked).

*This category included the subcategories of Straightforward/Easily

Understood 21 (61.8%), Informative 3 (8.8%), New Role/Perspective 3 (8.8%), and Other 7 (20.6%).

### Positives

Positive feedback was classified into three primary categories.

#### 1. New perspective and/or patient role

More than half of respondents felt the letter was a positive intervention method because it offered them a non-confrontational approach for initiating a discussion with their provider. Additionally, some patients stated it served as an instrument to engage them to be more active participants in their healthcare, evoking questions they otherwise wouldn't have thought to ask. Several patients' perceived the letter as a tool that 'empowered' them to take a role in the management of their hypertension.

'It referred me for questions that I should ask the doctor about and so forth, and, you know, I'd never give a thought about asking before.' (Arm B)

'Well, you know, it made me more aware of what I need to do, concerning my blood pressure. I believe, you know, and something else is that this letter empowered me more to do it, to tell you the truth.' (Arm B)

#### 2. Straightforward/easily understood

Additionally, several patients felt the letter was clear and easily understood, asking them to discuss with their providers the possibility of using a thiazide for their hypertension.

'It was pretty straightforward, just wanted me to talk to the doctor.' (Arm C)

'Oh I thought it was very simple and straight to the point. It wanted--it just wanted me to talk to him about if I--if he thought I needed that, and you know. I just thought it was very well explained letter. Didn't have any trouble with it at all.' (Arm C)

#### 3. Informative

Patients also saw the letter as positive because it provided them with information that was useful in understanding their hypertension and various treatment options.

'Well I think it was a reasonable request. I appreciate that information.' (Arm B)

### Negatives

Two patients offering negative feedback worried that the intervention challenged their providers' medical practice regarding prescribing behaviors. These patients were uncomfortable with the new role they were asked to adopt. At the same time, both of these patients chose to bring in the letter to their appointment to get their providers' opinions.

'Well, you know the way I looked at it right away was, they're telling me that I should tell the doctor I need to take it and I thought well, I really don't want to do that. I want him to tell me I should take it.' (Arm C)

'Well, I thought it might get my doctor a little shook up. I mean, thinking I'm trying to go over his head or something. I didn't want to do anything like that. 'Cause I like--I think he's a good doctor.' (Arm B)

The other two patients stating negative opinions felt the letter didn't take into consideration their co-morbid conditions.

### Motivations for bringing in the letter

In addition to patients' opinions of the intervention letter, we analyzed the motivational factors that encouraged those who brought the letter to their providers for discussion (n = 45) to do so (Table 3). Patients provided feedback as to what prompted them to follow-through with the intervention, with four primary themes emerging:

**Table 3:** Patients' motivation for bringing in letter

	(N = 45)^
**Motivation for patient bringing letter to appointment**	
Military/VA culture*	17 (37.8%)
Information seeking	12 (26.7%)
Changed patients' receptiveness to antihypertensive	2 (4.4%)
Just did it	4 (8.9%)
Other	6 (13.3%)

**Did not bring in the letter**	4 (8.9%)

Nine participants did not have complete question/response pairs (participant was not asked the question or did not directly answer it when asked).

*This category included the subcategories: following orders, 12 (70.6%); and serving others, 5 (29.4%).

#### 1. Sense of obligation

Many patients (37.8%) noted they brought the letter to their providers out of a sense of obligation, either following the directions of the letter because they were told to or out of a greater sense of paying back to fellow veterans and society.

##### Following orders

For most patients (70.6%), whose motivations were sub-coded as a sense of obligation, the idea of following instruction appears to be ingrained in their reasoning for bringing in the letter. These individuals stated they were simply doing what the letter requested of them.

'For one thing I was told and I listened.' (Arm A)

##### Serving others to give back

Another motivator within this category was the fact patients knew they were involved in a VA study. Five of the respondents reported they followed through with what the letter asked them to do because they wanted to be a part of the study to benefit other veterans. This altruism too may be part of a larger VA culture where many individuals seek to serve others or give back to society [29].

'This is kind of hard to explain. But I've had a heart transplant in the past like seven years ago, and before my transplant I was involved in a number of studies. And I just feel that's my way of paying back a little bit maybe.' (Arm B)

'I figured if you're doing a study there's a purpose for me to do all this stuff and, and, I don't have to know the reason necessarily, it's just that it was no big imposition on my part.' (Arm C)

#### 2. Information seeking

The second most prevalent rationale for bringing the letter to their provider was to glean additional information about alternative treatment options.

'Well, just to make sure I had all the facts correct.' (Arm C)

'Well, sometimes if you go to the doctor with a new idea, they think you've been on the internet reading some kind of hocus pocus thing that you got in the mail. I wanted to make sure that he [provider] realized it was part of a study and not just some cockamamie thing I'd come up with.' (Arm A)

Additionally, a subcategory within this group of responses suggested some patients brought the letter in because they were concerned about their hypertension along with a co-existing health condition, which they wanted to talk with their provider about in more depth.

#### 3. Changed patients' receptiveness to being prescribed an antihypertensive

For a small number of patients (4.4%), the information in the letter reinforced previous hypertension conversations they had with their providers and actually increased their willingness to try to reduce their high blood pressure through a prescribed medication.

'Right, I brought everything in and we talked about it. And we discussed it three months prior to that, but they thought I might be losing some weight, that my blood pressure might drop. So they waited to put me on medication. So they went ahead and put me on medication this time.' (Arm C)

'Well, the week before I went to mental health at the VA and they do blood pressure in there, you know, and of course I suffer from an anxiety disorder, so at the time I was really feeling a lot of anxiety and that, and she took my blood pressure, and it was like, 190/113. And that I hadn't really worried about it until I seen that [letter], and you know, I'm setting myself up for a heart attack or stroke or something, you know.' (Arm B)

#### 4. Just did it

The final reason patients offered for bringing in the letter for discussion was they just did it. Nine percent of the respondents answered in this noncommittal and less descriptive manner.

'Well, I don't know, I just thought I would. Just to see.' (Arm B)

### Why Patients did not bring in the letter

Of the four patients who reported not bringing in the letter, the reasons for their decision were vague, although they do provide some insight. Two didn't remember seeing the letter and two others mentioned their hypertension was controlled before or at the time of their appointment; therefore, they did not see a need to address the issue.

### Perceived reasons for why a patient was or was not prescribed a thiazide diuretic

Finally, we analyzed patient perspectives regarding why a thiazide was prescribed or not. Patients were asked during the semi-structured interviews what they believed was the main reason for whether they received a thiazide prescription. The reasons given by the patients are reported at an individual level and offer insight into patients' interpretations of their providers' prescribing rationale, and, for some patients, how they see their role in the decision-making process. A complimentary paper reports on an analysis of semi-structured interviews with providers and presents a more direct assessment of provider reasons for prescribing or not prescribing a thiazide [20].

### Patient perceptions of why they were prescribed a diuretic

Of the 50 patients who brought in the letter discussing their blood pressure, one-half of them were prescribed a thiazide diuretic (Table 4). Four themes were derived from patient responses regarding the reasons they believed they were prescribed a thiazide.

**Table 4:** Patients' perceptions of reasons prescribed thiazide diuretic (n = 25)

	Primary Reason Prescribed	Secondary Reason Prescribed	Combined Total
To Lower Their BP	12 (48.0%)	3 (27.3%)	15 (41.7%)

Because of Co-Morbidities	1 (4.0%)	4 (36.4%)	5 (13.9%)

To 'Try It'/Because it's a 'Good Idea'	10 (40.0%)	3 (27.3%)	13 (36.1%)

Because Their Doctor Knows Best	2 (8.0%)	1 (9.1%)	3 (8.3%)

	25	11	36

#### 1. Lowering blood pressure

The majority of those prescribed (48%) thought lowering their blood pressure was the primary reason they were prescribed a thiazide.

'Well, he [provider] wanted to lower my blood pressure another four or five points, something like that. It wasn't elevated too much, but it would be an advantage to bring it down some more.' (Arm B)

'Well I thought it was appropriate because my blood pressure has been too high. So the doctor concurred with your advice on using a diuretic.' (Arm A)

#### 2. Good idea

Many patients (40%) also mentioned they were prescribed a diuretic simply because their providers wanted to 'try it' or because the intervention 'sounded like a good idea.'

'Well, I had the card that said I should discuss, or would I discuss with the doctor, about the diuretic for my blood pressure, and the doctor said, 'Oh yes, I think that's a very good idea for you." (Arm B)

#### 3. Doctor knows best

Keeping with the paternalistic model of healthcare, a few patients (8%) mentioned it was their providers' decision whether they were prescribed a diuretic. Patients' stated they were unqualified to make healthcare decisions and trusted their providers to do what was best. These patients did not offer an explanation of their providers' prescribing rationale.

'I just thought I'd leave it in the hands of the doctor. I have faith in the doctor and he takes care of me.' (Arm C)

'Well, he said it was up to me basically and I knew that. I'm no authority on the list of medications and so I just went with what he thought would be the best for me.' (Arm C)

#### 4. Co-morbid Conditions

One of the patients stated they were prescribed a diuretic primarily because of a co-morbid condition; however, a few more listed co-morbidities as secondary reasons. The co-morbidities included edema or increased levels of potassium or creatinine.

'Well, I had a little bit of swelling in my legs at that time. When you pull the socks down you could see little indentations. You know, he said he really never checked for that before. He said, I had a little bit but not a lot, but he said maybe this would work and might bring it down a little more than what we had been doing.' (Arm B)

### Patient perceptions of why they were not prescribed a diuretic

Twenty-five patients were not prescribed a diuretic (Table 5). Four themes were derived from the responses regarding their belief as to why they were not prescribed a thiazide.

**Table 5:** Patient perceptions of reasons not prescribed thiazide diuretic (n = 25)

	Primary Reason Not Prescribed	Secondary Reason Not Prescribed	Combined Total
BP Currently Controlled*	9 (36.0%)	4 (33.3%)	13 (35.1%)

Because of a Co-Morbidity	8 (32.0%)	4 (33.3%)	12 (32.4%)

Intensified Therapy	5 (20.0%)	3 (25.0%)	8 (21.6%)

Side Effects	2 (8.0%)	1 (8.3%)	3 (8.1%)

Other	1 (4.0%)	0 (0%)	1 (2.7%)

	25	12	37

* Reasons patients gave for their bp currently being controlled included the doctor telling them their bp was ok 4 (44.4%), they liked the way things were going or 'didn't want to upset the apple cart' 2 (22.2%), they were taking enough meds 1 (11.1%), their blood pressure was too low 1 (11.1%), or they had 'white coat syndrome' 1 (11.1%).

#### 1. Blood pressure controlled

Approximately 36% of the patients who were not prescribed a thiazide said this decision was made because their blood pressure was controlled. Several patients apparently made the *a priori *decision not to be prescribed a thiazide, but brought in the letter anyway.

##### 'Let's not upset the apple cart'

One subgroup who made this *a priori *decision stated that they were satisfied with how their current regimen was working so they didn't want to make the change. At their primary care visits, they then presented their rationales to their providers.

'Well, I did take it to the clinic when I went last week. Gave it to 'em. Showed it to 'em. Frankly, I have tried--I've been on high blood pressure medication for approximately thirty years. So way back when, you know, [we tried] various deals including, the water pill, and finally we came up with this, Adelat [nifedipine] 60 milligrams. And, it's working great. So, I, told my doctor actually, I'd say PA, 'Well, unless you have, a great reason for changing, the [Adalat] is working good, let's not upset the apple cart,' and she agreed a hundred percent.' (Arm A)

'Well I wasn't too much in favor of changing anything because I felt my blood pressure was well-controlled. And so I was going to say, 'Here's this card, I don't think I want any changes." (Arm A)

##### White coat syndrome and home monitoring

Another subgroup told their providers they had 'white coat syndrome' and did not need to be prescribed a new medication because their own home blood pressure monitoring was evidence that their blood pressure was controlled.

'I told Dr. X that I had recorded a lot of my blood pressures in the year 2006 and I had that record and my pressure was consistently lower than the one that was recorded at the VA last time. I have been on diuretics before. I told him that in the history of my blood pressure I tried several different drugs prescribed by my local physician in order to try to get a handle on it and that I was satisfied with where I was at right now. I didn't feel that the pressure recorded at the VA was really the pressure that we should go by.' (Arm B)

#### 2. Co-morbid Conditions

Although this was also a reason given for being prescribed a thiazide, 32% of patients (not initiated on thiazide treatment) also described co-morbidity as a reason they were not prescribed a diuretic. Commonly reported comorbidities included benign prostatic hypertrophy (BPH) and diabetes.

'My doctor and I considered it [the letter], but presently I'm having a lot of kidney problems. I have been diagnosed with kidney disease, and they thought that at this time that it would not be a good idea. What they did instead was increase my blood pressure medicine a little bit. They were hoping that would take care of the problems I was having at the present time.' (Arm B)

#### 3. Intensified therapy, but not with a thiazide

A fifth of the patients for whom a thiazide was not prescribed stated that their provider chose to increase or add other therapies instead of initiating a thiazide diuretic. Over half of these patients said they were increased on their current therapy, while two were prescribed a different or additional drug. One patient had both their current prescription increased and another drug added to their regimen.

'I took it to the Dr. X at the VA hospital and he said that he just put me on a heavier, a stronger [dose of the] same medication, felodipine, and he said we'll see if that works and if that brings your pressure down a little bit then we'll put you on a diuretic. So he didn't shut it out, he just said we, he don't like to do that very well, apparently. And, that's ok with me. It's up to him.' (Arm C)

#### 4. Undocumented history of diuretic use

A few patients (n = 3) acknowledged they tried a diuretic in the past and had a side effect and asked not to be prescribed a diuretic. Although having a contraindication to diuretics was an exclusion criterion, we found it was often not documented in the patients' notes. Again, of note is their decision to bring in the letter despite knowing they did not want to be prescribed a diuretic.

'We had talked this over a year ago that I was on that kind of a pill, a fluid pill. And, it had got me to where I was peeing an awful lot. So, I quit taking it and told him I was going to quit taking it because I felt every fifteen minutes wasn't necessary and so he took me off of it then.' (Arm A)

Patients who were not prescribed a thiazide often had developed arguments for why they should not be prescribed, including documentation of home blood pressure readings and histories of previous diuretic prescriptions. At the same time, most had a positive view of the intervention and were appreciative of the conversation prompted by the letter.

## Discussion

The results presented indicate that a patient activation intervention was perceived by most patients as a positive and effective tool for increasing bidirectional interactions with their primary care providers and for implementing evidence-based guideline therapy. The acceptability of the intervention was demonstrated by the positive feedback received from a majority (83%) of the participants. For most patients, it was viewed as a straightforward tool to help them engage in conversation with their provider about information specific to the treatment of their hypertension. In addition, patients provided insight as to why the intervention was effective in increasing participation in the intervention. Many of their answers pointed toward findings consistent with previous studies that have established the efficacy of utilizing a collaborative approach to healthcare [3,10,14,30], which further reinforces the positive role patients can play in the promotion of guideline-based care of hypertension.

Patient responses detail some factors of patient activation interventions that appear to be important for their acceptability and effectiveness. First, providing patients with a straightforward, clearly written informational letter with a specific request offered them an opportunity to candidly discuss alternatives to treating their hypertension. Some patients went so far as to say it 'empowered' them or gave them permission to actively engage in conversation with their provider without being perceived as demanding. By having the letter in hand, patients' felt an added sense of validation for the queries they were presenting, which may have been particularly beneficial for patients who were reluctant to take a more active role in their interactions with their providers. A sizable portion stated they brought the letter in to become more knowledgeable regarding treatment options. Thus, the language of the intervention letter seemed to be expressed in a manner that was agreeable to both those who learned from the letter and those who used it as a means to learn more from their provider. These findings suggest that some patients may be hesitant to pursue detailed medical information from their provider--despite a desire--without aide from a trusted, external source, which well-designed patient activation interventions can provide.

The results from this paper and the parent study emphasize that most patients in the study were willing and interested in taking a proactive approach to their healthcare [21]. Other work by our group has shown that differences in patient role-orientation were independent of willingness to comply with the patient activation intervention [31]. In other words, patients who valued active engagement with their providers liked the intervention because it gave them a trusted tool to do so. On the other hand, patients who preferred to remain more passive in the clinical encounter also liked the intervention because they could just provide the information to their providers, while leaving the decision in their hands.

At the same time, it is clear that one size does not fit all when it comes to promoting patient engagement in healthcare. For a minority of patients, the intervention made them uncomfortable because they perceived the letter as questioning their provider's judgment. One way to ease the discomfort of some patients may be to specifically discuss the decision-making process in the letter, reassuring some patients that the intervention may encourage discussion between patients and providers, but ultimately the healthcare provider can make the final decision. Another approach could be to inform providers at the clinics of the letters and tell patients the providers are aware they may bring them to their visit. This could ease patients' discomfort if they know providers are prepared for the letters; however, this may also reduce the effectiveness of the intervention in prompting the providers. Future studies should address different ways of presenting clinical guideline information to patients--and providers.

The influence of military or VA culture was seen throughout patient responses. These findings are consistent with work conducted by Campbell and colleagues [29] regarding altruistic propensities of veterans in regards to volunteering for clinical trials. Their experiences with military culture and active duty service appear to embed core values such as altruism, stewardship, and a propensity to follow orders. Within the margins of this study, numerous patients' mentioned altruistic factors that influenced their receptiveness to and positive perceptions of the intervention. Responses also indicated a willingness to participate in the study and follow-through until completion with the goal of helping others. Additionally, many patients specified ethical motivations for participating in the study and for bringing in the letter for discussion. For several patients it was their way of 'paying back' the military and other veterans, while others were merely 'following orders', suggesting that some former service members still believe they have a duty to abide by direct orders.

These findings raise two issues for future implementation of this and similar interventions. First, would a patient activation intervention be as acceptable and effective in a non-VA population? Some veterans in this study stated they brought in the letter simply because it told them to do so. VA clinics may have patients who are more likely to comply with this type of intervention and the request to bring in the letter. However, as discussed previously, this patient activation intervention had appeal for a wide-variety of reasons beyond a sense of obligation and could potentially appeal to non-VA populations as well. The second issue is that the sense of obligation to participate in the intervention appeared to have increased because it was also a research study. This issue has important implications for implementation research as we seek to study the effects of an intervention in clinical practice--beyond the efficacy of a clinical trial. How do we separate the influences of participating in a study from the decision to participate in an intervention?

Finally, the results demonstrate the variety of roles patients played in the intervention. Some wanted to be informed healthcare consumers, including understanding why they are or are not prescribed a thiazide diuretic. Not all patients want to be active in the decision-making process. Some want to be informed about why they are receiving certain treatments, while others relied on the adage 'my doctor knows best.' Interestingly, even those who did not want to be part of the decision-making process still brought in the letter to have the conversation with their provider. Therefore, the emphasis on bidirectional interaction is not only about patients who want to be involved in the decision-making process, but also for patients who want to be informed--or to simply comply. The responses can also be compared to provider responses regarding why they chose to prescribe a thiazide to their patients [20]. The comparison may provide insights into the barriers to prescribing thiazides as a first-line therapy for hypertension. These barriers include the interaction between both stakeholder groups. For example, some patients in the study come in with their home blood pressure readings--and an argument for why they should not be prescribed a thaizide.

### Limitations

There are several limitations of this study. First, the study was restricted to a sample of predominately white male VA patients. The findings may be unique to the VA, as many veterans appear to exhibit a sense of obligation that may influence their participation in and perception of the interventions. This also raises questions for implementation research, which seeks to understand the effects of an intervention outside of research contexts. Secondly, we only interviewed four patients who did not discuss the letter with their providers. The choice to focus on the main outcome of the parent study (prescription of a thiazide) limited our ability to examine why patients chose not to bring in the letter; however, prescription of a thiazide was the most timely and reliably documented outcome by which to stratify for the qualitative sample. The likelihood is that the parent study actually underestimates the number of people who brought in the letter, and that the qualitative study over-emphasizes the acceptability of the intervention to patients. Another methodological limitation is that it is difficult to interpret the influence of the separate intervention arms due to our decision to collapse in the presentation of findings. However, based on an analysis of the matrix coding by arm of intervention, the arm of the patient's participation does not appear to affect their evaluation of the letter or patients' motivations for bringing in the letter. Equal numbers of patients who were prescribed a thiazide and who were not prescribed a thiazide were recruited in each arm, so it is difficult to evaluate qualitatively whether there were systematic differences by arm. It also illustrates the 'messy' nature of real-world implementation and the inability to capture every scenario to describe success or failure of an intervention. Nevertheless, we included the arm with each of the quotes for the reader's interpretation, although we recognize the readers do not have the advantage of seeing the depth of quotes and their consistency across arms. Finally, as noted in the tables, several participants' answers were missing. In large part, this is due to our decision to restrict matrix coding only to very specific question/response segments. The missing answers do not appear to be systematic.

## Summary

The primary purpose of this study was to evaluate the acceptability of a patient-activated intervention from the patients' perspective. Patients along a spectrum of role orientations appreciated the intervention as a trusted tool to engage their providers in an informed discussion about hypertension treatment options and clinical guidelines. Insight into how patients perceived the intervention strategy may serve to assist in the design of future low-cost interventions to improve the management of chronic diseases in VA and other health systems and have potential value to clinical administration leaders who are responsible for improving the quality of care. The patient activation strategy was acceptable to most patients, served as a tool to engage patients in a more active role, and seemed to promote greater patient-provider interaction.

## Competing interests

The authors declare that they have no competing interests.

## Authors' contributions

SAP participated in the qualitative analysis and prepared the draft of the manuscript. MBW participated in the design of the interview guide, conducted interviews, performed qualitative analysis, and contributed to drafting the manuscript. RHB participated in the design of the interview guide, conducted interviews, performed the qualitative analysis, and reviewed a draft of the manuscript. RG performed qualitative analysis and reviewed a draft of the manuscript. MVW and AJC contributed to the design of the study and reviewing and revising the manuscript. PJK was the principal investigator of the parent study and contributed significantly to the design of this study and conceptualizing, editing, and revising the manuscript. HSR oversaw the qualitative components of the parent study. For this paper, she coordinated the design of the study, conducted interviews, coordinated the analysis, and contributed significantly to conceptualizing, drafting, and revising the manuscript. All authors read and approved the final manuscript.
